# Care of the Teeth

**Published:** 1862-08

**Authors:** J. Taft


### CARE OF THE TEETH.

BY J. TAFT.

(Continued from page 306.)

FOR tooth-picks there is, perhaps, nothing better than the quill, though fine-grained, hard, tough wood is very good, but not so durable as quill. There is no liability of injury to the parts by the use of either of these.

Picks should always be thoroughly used after every meal, and in such a manner as to remove every particle of food from between the teeth. After the pick the floss silk, tape or rubber should be used.

Forcing water swiftly through between the teeth will remove loose particles of food that may be lodged upon and under the margin of the gums. Care should be exercised in the use of the silk, tape or rubber, lest the attachments of the teeth should be too much impinged upon.

The question is often asked, "What kind of a brush should I use?" and "can I use it too much?" To the first of these the reply is, that a brush should be as firm and stiff as the gums will tolerate; it should not irritate them by the use that is necessary to keep the teeth clean. The gums, and not the teeth, should determine the character of the brush, for no ordinary tooth-brush will injure the teeth, however much it may be used upon them. When the teeth are clean, the object is accomplished, and the brush has then performed its work for the time being.

In regard to dentifrices, or preparations for the teeth, we would suggest, that when they can readily be kept clean, and the surrounding parts healthy, nothing of the kind is required. If care and pure water, with the implements suggested, will accomplish the object, it is better than to use any preparation; and doubtless more attention and work would render the demand far less for tooth powders, pastes and washes.

These preparations are employed for two or three objects. One is to cleanse the teeth. Another is to medicate the surrounding tissues when in a state of disease. Another is to arrest decay of the teeth—the latter, however, is only indirect. The first is accomplished chiefly upon one principle, viz., by mechanical action, and by this only. This action, of course, should not be equally heroic in all instances, but should be regulated according to the requirements of the case, some requiring much more than others. In no case should any preparation be used for cleansing the teeth that will act chemically upon them. Many of the preparations sold for cleansing the teeth operate in this way, and in this consists their alleged merit; they should all be avoided.

The lotions or washes for the teeth and mouth are usually only efficient in correcting vitiated conditions of the secretions, and do not amount to much for the teeth; there are some, however, which contain some saponacious material—these serve well to wash the teeth. It is not an easy matter to give specific directions to those who have either general or local diseases that exercise a pernicious influence upon the teeth; the difficulty does not arise so much from deciding what should be done in any given case, as from the great variety of circumstances in which different cases are involved.

But two or three things may be suggested. And first, always when laboring under disease, of whatever character it may be, keep the teeth scrupulously clean; be more careful of them, if possible, than during good health. If the secretions are vitiated, so that they will act upon the teeth injuriously, something should be done to correct the difficulty. If there is an acid state of the secretions, some alkaline prepations, as a solution of bicarbonate of soda or prepared chalk, should be used three or four times a day, and sometimes more frequent still.

Special attention should be given at such times to prevent irritation or disease in the surrounding tissues ; this is liable to occur if there has been a predisposition. The care of the teeth during sickness is a subject that is very much neglected by both physician and patient; and it is perhaps more necessary to impress this fact than to specify definitely what should be done; for patients and nurses, if they feel the full importance of keeping the teeth in a good condition during sickness, will devise some method to accomplish it.

The brush should be used very thoroughly with soap, or some simple dentifrice.

The welfare of the teeth requires that they be used in a legitimate way, not only a portion of them, the teeth of one side for instance, but all should be used alike in the performance of their proper function. Many persons are in the habit of masticating all their food with the teeth of one side; this overburdens the teeth of one side, especially in respect to wear, while the teeth on the opposite side, being idle, are far more liable to become diseased ; no organ, thrown out of use, retains its normal strength and vigor. This applies to the teeth as surely as to any other organ. Hence, we insist that all the teeth should be used alike.

Perhaps nearly all the suggestions we have made in regard to the care of the teeth are familiar to a great majority of persons, so that in this, as in a great many other things, they do not put in practical exercise the knowledge they possess, and perhaps it would be better to endeavor to induce them to put in exercise that to which they have attained, rather than to be leading on to new ideas, that shall be disregarded, just as the old have been.

Occasionally a person is found, who says, "I have good teeth, and I never gave any attention to them; they have never been in the hands of a dentist; they have never ached, and they do their work well, and I think this special attention of the teeth is all nonsense." Now, for every such case, where the teeth are really sound, there will be found five hundred cases, where the teeth to a greater or less extent are lost, by pursuing just the course this person has done, simply neglecting the teeth. By the same kind of reasoning, every man, woman and child might become a brandy-guzzler, because one man in ten thousand can soak himself with brandy, from day to day, and become completely saturated with it, without becoming a gutter drunkard.

The only safe method is to use the teeth as nature intended they should be used, and in addition to this, keep them in the best possible condition, both in regard to cleanliness and health. Keep them beautiful, untarnished gems, that shall glow with vitality.

